# Premature ventricular contractions originating from the right ventricular outflow tract induced by adenosine triphosphate administration

**DOI:** 10.1016/j.hrcr.2026.05.008

**Published:** 2026-05-15

**Authors:** Yuki Kato, Yasuteru Yamauchi, Yuichiro Sagawa, Kazuya Murata, Kazutaka Aonuma, Tetsuo Sasano

**Affiliations:** 1 Department of Cardiology, Japan Red Cross Yokohama City Bay Hospital, Yokohama City, Kanagawa, Japan; 2 Department of Cardiology, Mito Saiseikai General Hospital, Mito, Ibaraki, Japan; 3 Department of Cardiovascular Medicine, Institute of Science Tokyo, Bunkyo-ku, Tokyo, Japan

**Keywords:** Premature ventricular contraction, Adenosine triphosphate, Ablation, RVOT, Difficult induction

**Key Teaching Points**

• Intravenous adenosine triphosphate (ATP) can provoke premature ventricular contractions (PVCs) when conventional induction methods such as isoproterenol or edrophonium fail, allowing successful mapping and ablation.
• ATP-induced PVCs exhibited a morphology consistent with a right ventricular outflow tract (RVOT) origin, enabling accurate localization through pace mapping despite limited activation mapping.
• In cases where spontaneous PVCs are absent in the electrophysiology laboratory and neither isoproterenol nor edrophonium induces a single PVC, ATP may serve as a useful alternative induction agent and should be considered as an option during the procedure.

## Introduction

Catheter ablation is considered a curative therapy for drug-resistant premature ventricular contractions (PVCs). However, the procedure can become challenging when PVCs cannot be induced during the electrophysiology study. In this case, although outpatient Holter monitoring documented PVCs and nonsustained ventricular tachycardia (NSVT), neither NSVT nor even a single PVC appeared in the laboratory. Intravenous adenosine triphosphate (ATP) provoked reproducible PVCs, which were then targeted using a pace-mapping approach. Ablation at the matched site successfully eliminated the arrhythmia, achieving a complete resolution.

## Case report

A 49-year-old woman with no significant medical history presented in 2019 with a sudden syncopal episode without prodromal symptoms or preceding dizziness. Evaluation at a local cardiology clinic via Holter monitoring revealed episodes of NSVT. Carvedilol (3.75 mg/d) was initiated, which reduced her presyncopal symptoms, although they did not completely resolve. She was subsequently referred to our outpatient clinic for further evaluation.

**Figure 1:**
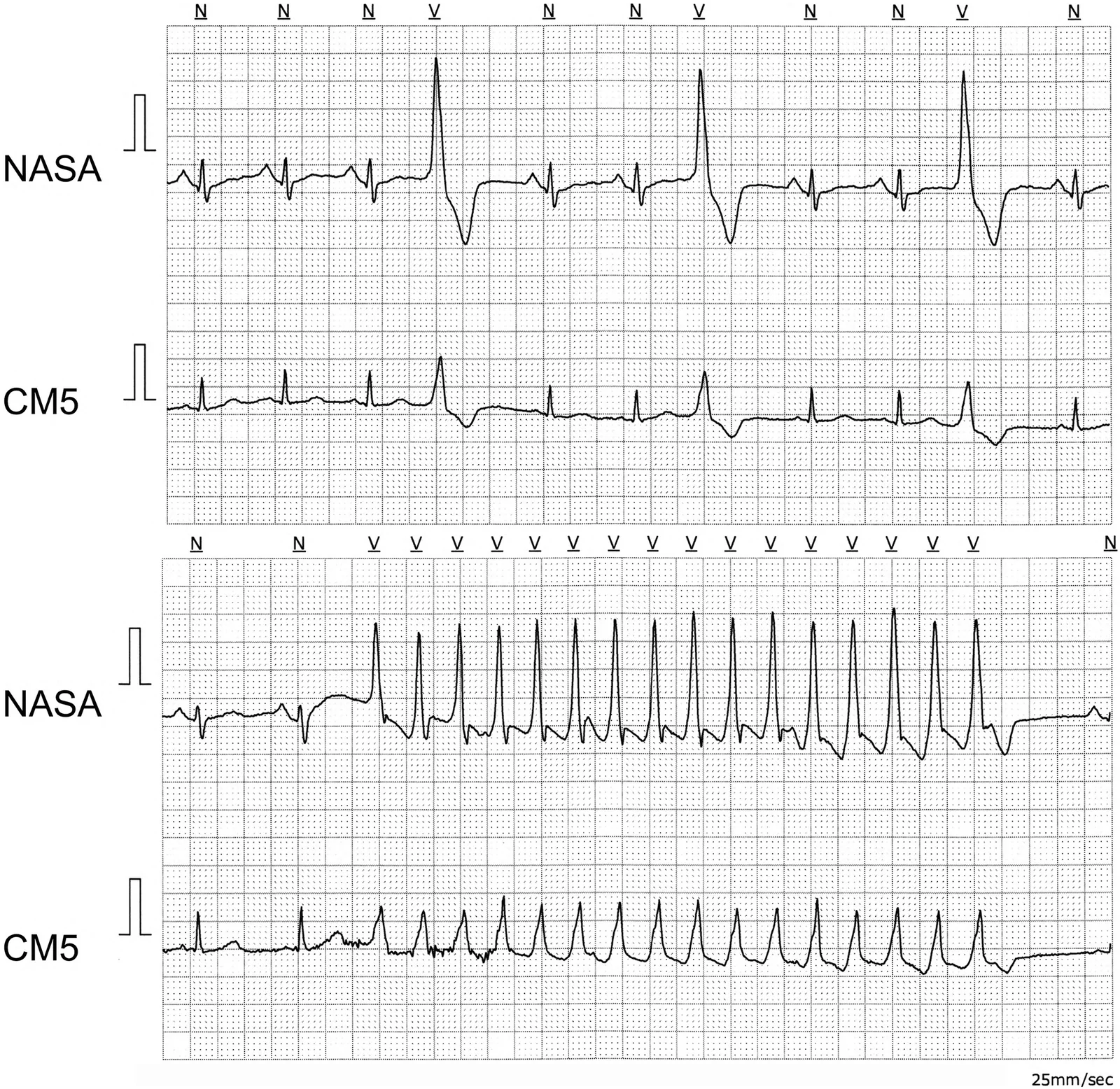
Holter monitoring. Based on the NASA lead and CM5 lead morphologies, premature ventricular contractions were suspected to originate from the outflow tract.

Repeat Holter monitoring at our institution showed a high burden of PVCs, accounting for 7.2% of total daily beats, along with occasional NSVT episodes. The 2 leads on the Holter electrocardiogram suggested a possible outflow tract origin of the PVCs and NSVT (Figure 1). The longest run of NSVT consisted of 16 consecutive beats, and no clear diurnal variation was noted. Transthoracic echocardiography revealed a preserved left ventricular ejection fraction of 63% and no significant structural or valvular abnormalities, so cardiac magnetic resonance imaging was not performed. A 12-lead electrocardiogram failed to capture any PVCs, making precise localization of their origin difficult.

**Figure 2:**
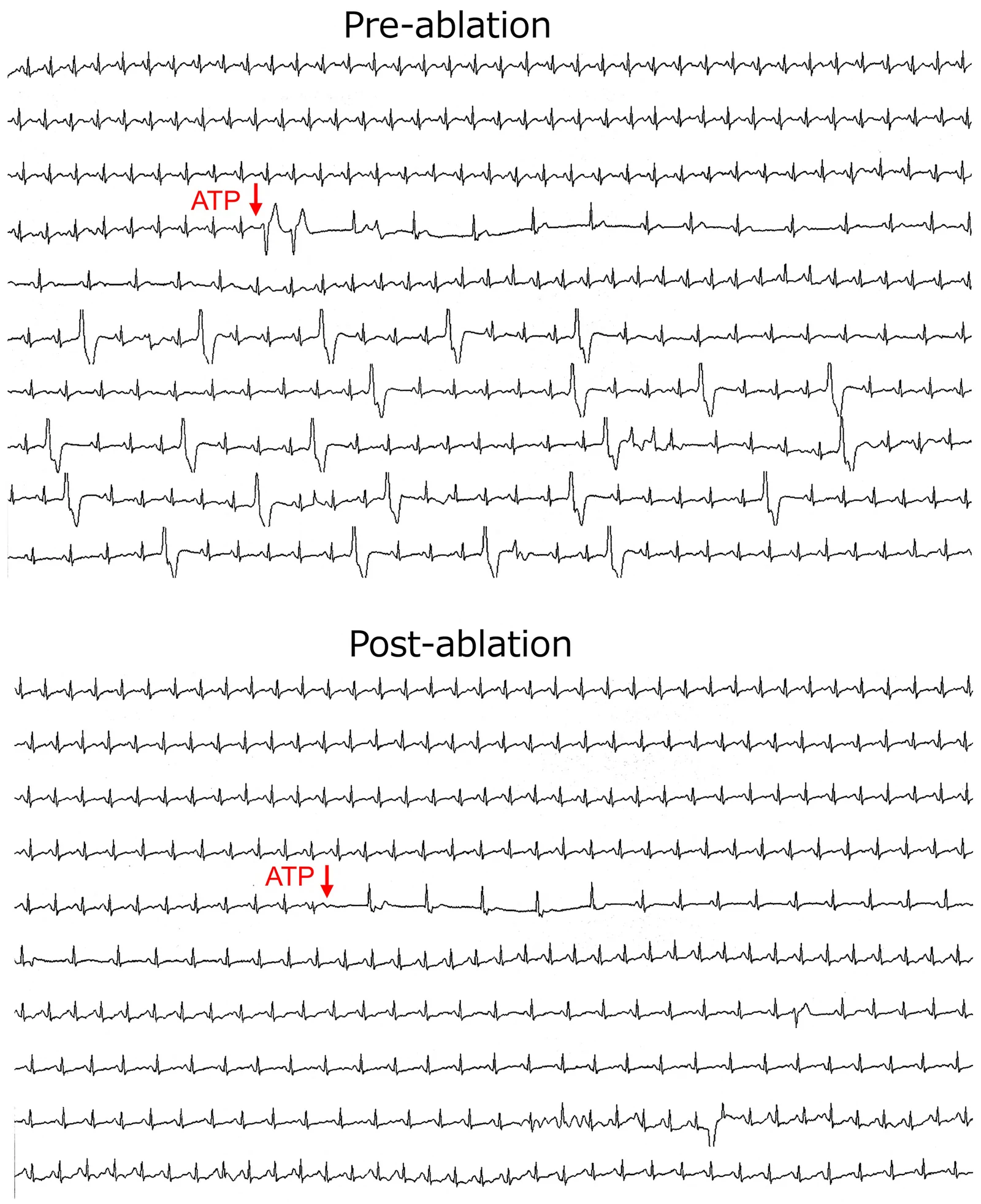
Electrocardiogram recording during the procedure. **A:** No premature ventricular contractions (PVCs) were observed at the beginning of the procedure, and neither isoproterenol nor edrophonium induced any PVCs. However, approximately 30 seconds after the administration of adenosine triphosphate (ATP), sinus arrest or bradycardia developed, followed by the intermittent appearance of PVCs. **B:** After ablation, the same intravenous administration of ATP no longer induced any PVCs.

**Figure 3:**
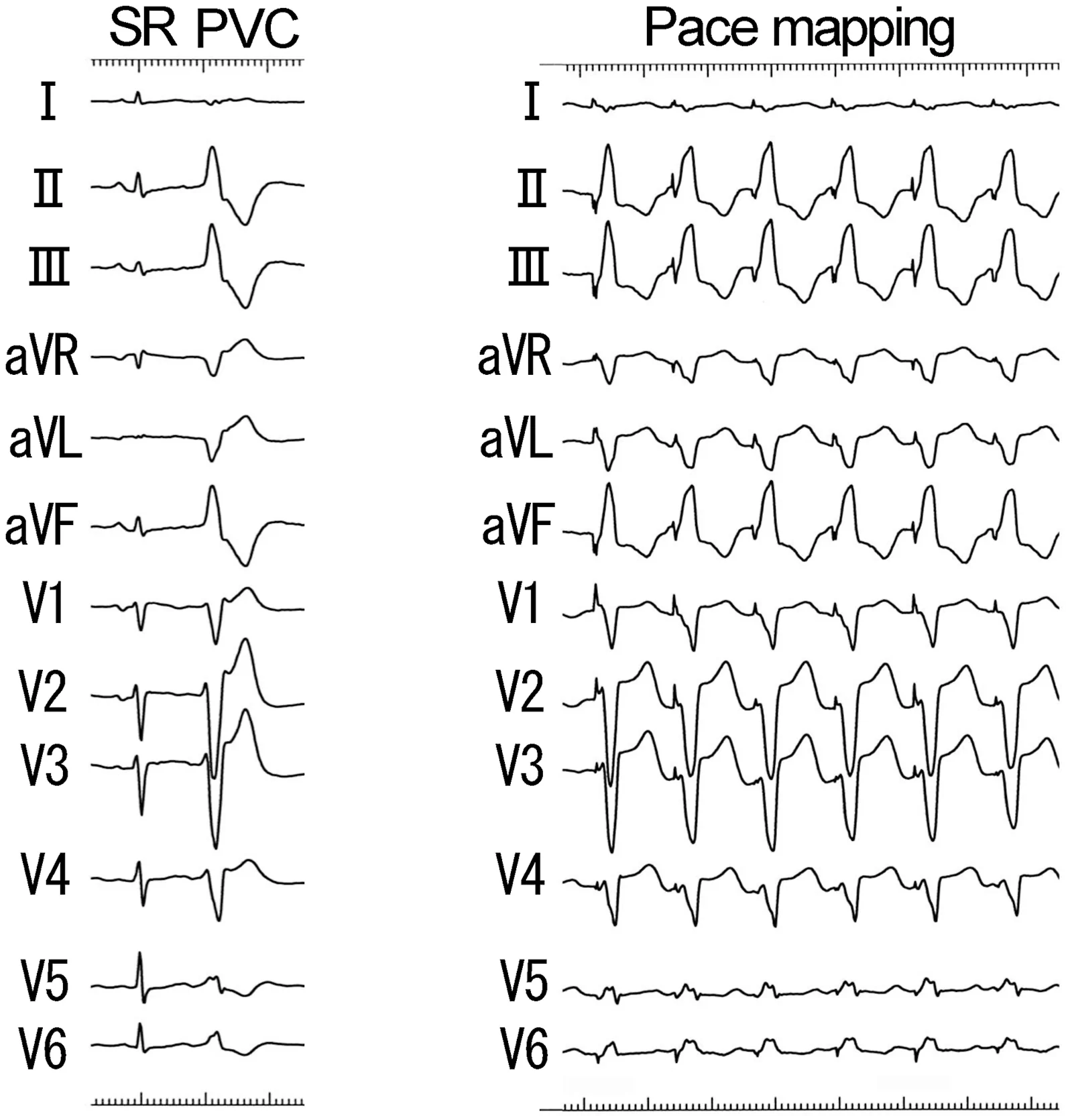
12-Lead electrocardiogram of premature ventricular contractions (PVCs) induced by intravenous adenosine triphosphate (ATP) administration and pace mapping. PVCs induced by ATP showed tall R waves in the inferior leads, and the precordial transitional zone was located between leads V_4_ and V_5_, suggesting an origin in the right ventricular outflow tract (RVOT). Pace mapping at the anteroseptal region of the RVOT produced the best pace-mapping score, yielding a morphology that closely matched the clinical PVCs. SR = sinus rhythm.

**Figure 4:**
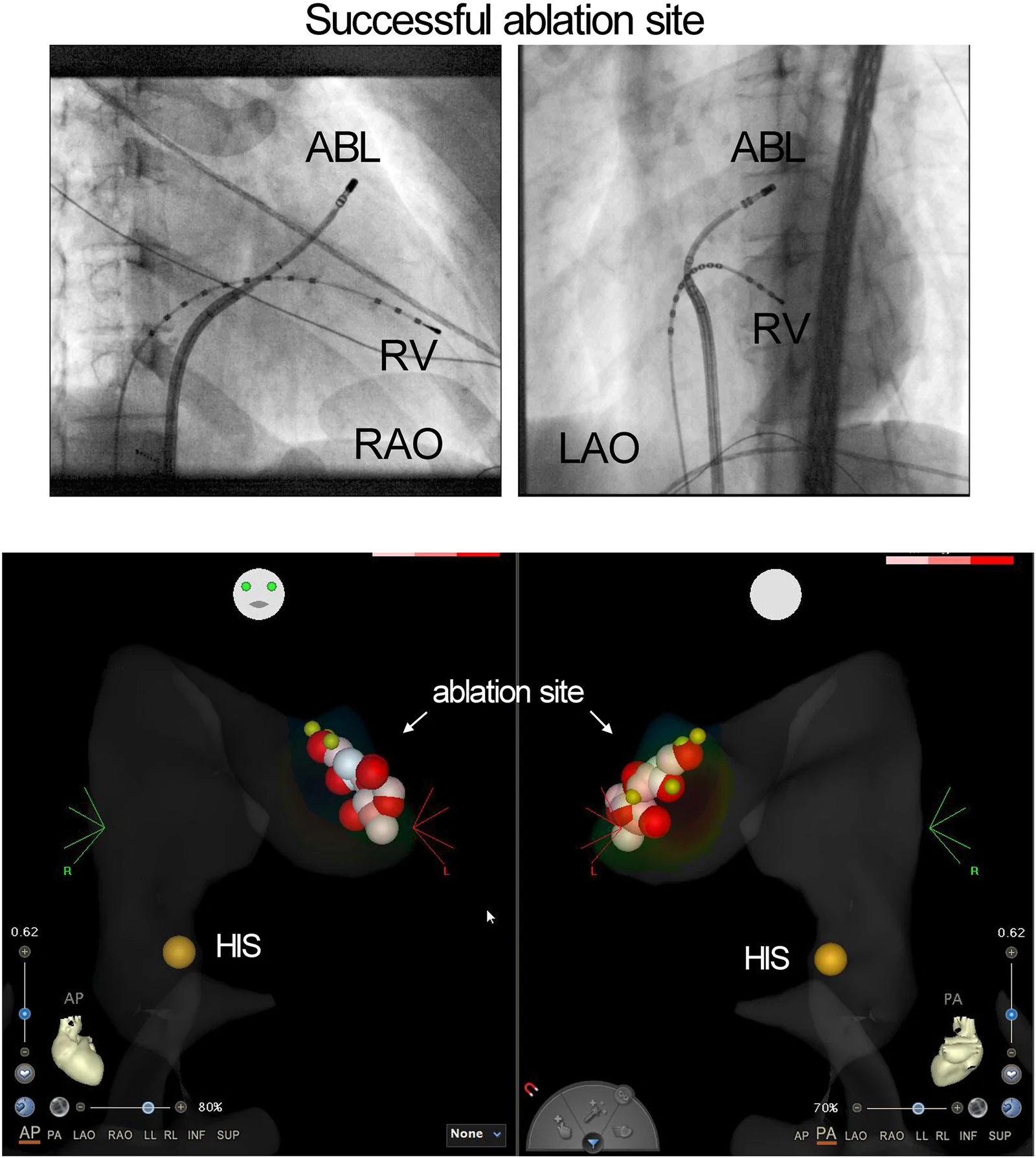
Successful ablation site and activation mapping. The fluoroscopic images showing the successful ablation site are presented in the upper panel, and the corresponding 3-dimensional mapping images are shown in the lower panel. Radiofrequency applications delivered several times at the anteroseptal region just below the pulmonary valve, as identified by pace mapping, successfully eliminated premature ventricular contractions (PVCs). The *white arrow* indicates the site of ablation. ABL = ablation catheter; HIS = His bundle; LAO = left anterior oblique; RAO = right anterior oblique; RV = right ventricle.

Given the clinical context, catheter ablation was planned for symptomatic PVCs/NSVT. However, upon entering the electrophysiology laboratory, no spontaneous PVCs were observed for approximately 45 minutes. Administration of isoproterenol (ISP) failed to provoke the arrhythmia. Also, edrophonium was administered, but it was ineffective. Subsequently, intravenous ATP (20 mg) was administered once, which successfully induced clinical PVCs. The latency from ATP injection to PVC onset was approximately 30 seconds (Figure 2A). These ATP-induced PVCs were observed transiently during the reflex tachycardia phase subsequent to sinus arrest. PVCs exhibited a left bundle branch block morphology, an inferior axis, and a precordial transition zone between leads V_4_ and V_5_, consistent with a right ventricular outflow tract (RVOT) origin. We used the electroanatomic mapping system (CARTO 3, Biosense Webster, Irvine, CA). The site of origin of the PVC was determined using detailed activation mapping and pace mapping. Activation mapping revealed that the RVOT region was the earliest site of activation. However, PVCs again disappeared during the procedure, which prevented further precise activation mapping. Therefore, ablation was primarily guided by pace mapping. Pace mapping demonstrated the best match at the anteroseptal aspect just below the pulmonary valve (Figure 3). Radiofrequency ablation at this site, along with additional applications over a broader surrounding area, was performed with a power setting of 30 W for 45 seconds per application with irrigation (Figure 4). Pace mapping at the successful ablation site demonstrated a close match to the clinical PVC morphology (96% match). After ablation, PVCs were no longer inducible with ISP or ATP provocation (Figure 2B). Because spontaneous PVCs were not sufficiently frequent to allow detailed activation mapping, the procedure was primarily guided by pace mapping. A distinct earliest local electrogram preceding QRS onset was not consistently identified. Radiofrequency energy was delivered at and around the site demonstrating the best pace-mapping match, which successfully eliminated the clinical PVCs. Postoperative Holter monitoring demonstrated a marked reduction in PVC burden to less than 0.1% of total beats, with complete elimination of NSVT. The patient’s symptoms resolved entirely.

## Discussion

We encountered a case in which ATP successfully induced PVCs that had been otherwise difficult to elicit. Although the overall PVC burden was relatively modest (7.2%), the patient exhibited NSVT at a rate of approximately 210 beats/min. While the documented episodes were self-terminating, even brief runs at this rate may significantly compromise cardiac output and lead to transient cerebral hypoperfusion. A slightly longer episode of such tachycardia could reasonably result in syncope. In the present case, the syncopal episode occurred in temporal association with rapid ventricular arrhythmias, and no alternative cause was identified despite comprehensive evaluation. Furthermore, elimination of the clinical PVCs and NSVT after catheter ablation was associated with the absence of recurrent syncope during follow-up. An implantable loop recorder was not implanted because syncope did not recur during follow-up.

Ertan and colleagues^1^ reported cases in which administration of ATP to terminate supraventricular tachycardia induced PVCs or NSVT after tachycardia termination. These ventricular arrhythmias induced by ATP were considered clinically insignificant in the absence of underlying heart disease. To our knowledge, although ventricular arrhythmias including PVCs and NSVT have been reported after ATP administration,^1^ the deliberate use of intravenous ATP as a provocative agent to reproducibly induce idiopathic PVCs for mapping and ablation remains rarely described. In our case, ATP administration successfully induced clinical PVCs, enabling precise localization of the origin and subsequent catheter ablation. In PVC ablation procedures, the absence of spontaneous PVCs occasionally results in procedure cancellation.

ATP is known to induce transient sinus bradycardia or sinus arrest via adenosine receptor activation, which may subsequently lead to autonomic rebound and increased sympathetic tone. Idiopathic RVOT PVCs are typically catecholamine-sensitive and are thought to be mediated by triggered activity, often related to cyclic adenosine monophosphate-dependent delayed afterdepolarizations.^2^ Therefore, in the present case, ATP administration may not have directly triggered PVCs but may have indirectly facilitated their induction through autonomic modulation and sympathetic rebound after transient bradycardia/sinus arrest.

This case suggests that ATP may serve as a useful pharmacological option for inducing PVCs. While adenosine is generally considered to suppress induced ventricular tachycardia,^3^ it can also promote catecholamine release and enhance sympathetic activity via stimulation of chemo- and baroreceptors. This paradoxical effect may enhance focal automaticity or triggered activity, thereby facilitating arrhythmogenesis.^4^ Previous reports have shown that ATP administration for the termination of supraventricular tachycardia resulted in PVCs in approximately two-thirds of cases, with most originating from the basal left ventricular septum. In contrast, PVCs in the present case originated from the RVOT, differing from prior reports and representing a novel finding.^5^

Regarding the induction of PVCs, Hachiya and colleagues^6^ have also reported the usefulness of edrophonium for inducing PVCs, particularly in cases in which vagally mediated PVCs occur more frequently at night. In this case, PVCs did not exhibit a nocturnal predominance and occurred consistently throughout the day. Although 5 mg of edrophonium was administered intravenously according to the protocol described by Hachiya et al as an alternative provocative maneuver, it was ineffective, and ISP also failed to induce PVCs. In the present case, there were no specific clinical clues in the patient’s history suggesting that ATP would be particularly effective for PVC induction. ATP was selected empirically on the basis of its known autonomic effects and its frequent use in the electrophysiology laboratory. Nonpharmacological maneuvers (eg, postural changes) were not attempted in this case. Without successful PVC induction by ATP, catheter ablation would likely not have been feasible for this patient. In cases in which PVC induction is difficult, agents such as ISP, edrophonium, adrenaline, and phenylephrine have been reported as potential options. The present case suggests that ATP should also be considered as an additional pharmacological tool for PVC induction.

## Conclusion

ATP can serve as a practical alternative induction agent when PVCs cannot be provoked by conventional methods, thereby facilitating successful mapping and ablation.

## Disclosures

The authors have no conflicts of interest to disclose.
